# The Professional Development Needs of Hospital Teachers in Ireland: An Exploratory Case Study

**DOI:** 10.5334/cie.123

**Published:** 2024-04-15

**Authors:** Fergal McNamara

**Affiliations:** 1 Solas Hospital School, City of Dublin Education and Training Board, Ireland

**Keywords:** Hospital school, hospital teacher, children with medical needs

## Abstract

In Ireland, hospital schools comprise a very small component of the overall primary and post-primary education system. Indeed, there are only seven hospital schools employing a total of 21 teachers nationally. Given the size and uniqueness of this education setting, opportunities for teacher continuous professional development are limited. This qualitative research study examined Irish hospital teachers’ perceptions of their continuing professional development needs using a case study approach. The research captured the perspectives of 19 teachers currently teaching in seven hospital schools in Ireland. Data consisted of responses to an anonymous online questionnaire and two focus groups designed to examine and interpret the questionnaire data. Thematic analysis was conducted on all data collected.

The findings revealed that Irish hospital teachers have a clear shared perception of the professional development needs both for newly hired and currently practising hospital teachers. Hospital schools are a very small, but a very important component of education in Ireland, and the professional needs identified are specific to this unique context. Teachers working in this context must be supported so that they can provide the education that the pupils, who must access this service, deserve.

Internationally, hospital schools are found in most inpatient paediatric services and are well established (Benigno & Fante, 2020; Capurso & Dennis, 2017). They provide for the education of children and young adults who are hospitalised, either in one-to-one sessions at the bedside or in small multi-level classrooms within or adjacent to a paediatric hospital or ward (Avalos & Fernandez 2021; Harris & Farrell, 2004; Keehan, 2021).

In Ireland, hospital schools have been in existence since the early twentieth century and formally recognised by the Department of Education since the 1960s (Board of Management of Our Lady’s Hospital Special School, 1964; Kennerk, 2019). They are categorised as special schools in the Key Statistics National School Census as issued by the Department of Education (DES, 2021).

Special education in Ireland is underpinned by two key documents; The Special Education Review Committee Report in 1993 (DE, 1993) and Education for Persons with Special Educational Needs Act 2004 (EPSEN Act, 2004). Both of these documents define special educational needs broadly and include children who are educationally disadvantaged due to their medical needs. While Irish hospital schools operate under the special education section of the Department of Education, they are not listed as special schools with the National Council for Special Education (NCSE, 2022).

Currently 11 public hospital schools are recognised by the Department of Education – four operating in Child and Adolescent Mental Health Service (CAMHS) inpatient units and seven within paediatric hospitals or wards across the country (DES, 2020, 2023). Teachers working within these hospital schools do not need a special qualification beyond that of a mainstream teacher in the Irish school system.

This study focused on the seven schools operating within paediatric hospitals or wards as they have been grouped together by the Department of Education report (DES, 2023). They share a pupil-teacher ratio of 10 to 1, based on the special school category of physical disability. The official number of pupils in a given school is determined by an annual census conducted on the last working day of September which then determines the staffing level. At the time of the study, the seven schools employed a total of 21 primary and post-primary qualified teachers.

Irish hospital schools exist as third-party organisations separate from the hospital sites that they serve. While they operate under the direction of the Department of Education, however, they also must operate within a larger organisation that is under the remit of the Department of Health. As such, hospital teachers occupy a liminal space as they operate as a part of the hospital team but are not the employees of the hospitals within which they must work.

As a relatively small component of the overall primary and post-primary education system, few teachers in Ireland have experience teaching in this milieu. Furthermore, for those who move from mainstream or special education settings into this unique context, there is no context-specific training available. This represents a significant challenge to the leadership teams in hospital schools. For example, when new staff start there is no agreed-upon induction plan or pathway to support them. In addition, due to staffing procedures in the schools, there is often no time assigned to the induction of staff, yet teaching must begin on day one.

Not surprisingly, it is recognised in the international literature that there is a need for context-specific training for teachers working in this environment (McNamara, 2022). Given the paucity of research into the professional development needs of hospital teachers, both internationally and especially in the Irish context, this exploratory case study aimed to identify the perceived professional development needs of Irish hospital teachers.

## Preliminary Survey of the Literature

A scoping review of the academic literature relating to this topic was conducted prior to the design of this case study (McNamara, 2022) looking at original research published in peer-reviewed journals between January 2011 and April 2021. Systematic searches were conducted on the following electronic databases: Academic Search Complete, British Education Index, Education Research Complete, SAGE Journals, Scopus, SpringerLink and Taylor & Francis Journals. A number of possible professional development topics were identified by this literature review and are summarised below.

### Context-Specific Training

All of the evidence studied agreed that hospital teaching presents a unique set of challenges and requires a specific set of skills. Several studies pointed out that there is a significant lack of context-specific learning pathways on offer to hospital teachers (Benigno & Fante, 2020; Benigno et al., 2018; Hen 2020; Keehan, 2021; Shore, 2019). A number of these studies highlighted the possibilities of psychological risk that this places on teachers (Benigno & Fante, 2020; Hen, 2020; Shore, 2019).

Only one study, Benigno et al. (2018), that surveyed Italian hospital teachers looked to begin formalising a context-specific professional learning pathway for hospital teachers, specifically a curriculum combining online training with interactions in a professional community of hospital teachers. The beginnings of this pilot project were well received by the hospital teachers in their study (Benigno et al., 2018). The idea of utilising professional communities of learning as a component of professional development is echoed in Keehan’s (2021) study of hospital teaching provision and the work of hospital teachers in Ireland.

### Knowledge of Specific Medical Conditions

One of the most apparent knowledge gaps between mainstream and hospital teaching is knowledge of specific medical conditions. For the hospital teacher, there is an identified need for professional development on how certain medical conditions or treatment regimes might impact the students’ ability to learn (Benigno & Fante, 2020; Benigno et al., 2018; Harris & Farrell, 2004; Keehan, 2021; Shore, 2019). Such information can then be used by the hospital teacher to tailor learning opportunities effectively to individual students (Keehan, 2021; Shore, 2019).

### Emotional Intelligence and Self-Care Skills

A number of researchers have proposed that the greatest challenge for hospital teachers is the emotional labour that is associated with working in this context (Hen, 2020; Keehan, 2021; Shore, 2019). As a result, they advocate for training to ensure teachers develop their emotional intelligence as well as interpersonal and self-care skills to help enable them to cope with their own emotions and to empathise with the emotions of their students and their parents (Benigno et al., 2018; Hen, 2020; Keehan, 2021; Shore, 2019).

The findings of one study (Hen, 2020), that looked at providing hospital teachers with an academic course in emotional intelligence reinforced the view that academic courses and training in emotional intelligence skills should be part of the professional development of hospital teachers.

### Digital and Mobile Technology

Two research studies focused on the importance of digital and mobile technologies for the professional development of hospital teachers (Maor et al., 2020; McCarthy et al., 2017) recognised that it is more challenging to implement these types of intervention in complex environments such as those of hospital teaching. As a result, they stressed that any professional learning, digital or otherwise, must take the context into account and therefore needs to be specifically tailored to hospital schools (Maor et al., 2020; McCarthy et al., 2017).

### Coaching

Both Maor et al. (2020) and McCarthy et al. (2017) propose coaching as the methodology best suited to providing professional development opportunities in complex contexts such as that of the hospital teacher. Coaches can assess the real-world challenges of the working environment and work with teachers on site to personalise training to individuals and tailor learning experiences to fit the context (Maor et al., 2020; McCarthy et al., 2017; Perry et al., 2014).

## The Present Study

Following on from the case study conducted by Keehan (2021), which looked at the provision for education and the challenges met by hospital teachers in Irish hospital schools, and from a scoping review of the literature investigating professional learning opportunities for hospital teachers (McNamara, 2022), the present study examined the perceived training and professional development needs of hospital teachers in Ireland.

## Method

### Design

The following two research questions underpin this project:

1. What professional development is required to enable qualified teachers to succeed as hospital teachers in Ireland?
2. What continuing professional development is required by Irish hospital teachers to allow them to reach their full potential as hospital teachers?

To address these questions a qualitative research strategy was chosen to explore Irish hospital teachers’ perceptions of their professional development needs. In particular, a case study approach was used as it allows for a number of data collection methods to be employed to fully elucidate the topic under examination (Merriam & Tisdell, 2015). Given the lack of research in an Irish context and the limited population of Irish hospital teachers, this approach was deemed appropriate (Zainal, 2007).

The goal of the case study was to capture the perceptions of hospital teachers currently employed in Irish hospital schools. It is, therefore, bounded both geographically and temporally. Case studies are not designed for creating generalisable findings but rather to examine a specific case from which other similar cases might find inspiration (Creswell, 2013; Creswell & Cresswell, 2018; Gerring, 2017; Merriam & Tisdell, 2015).

The study involved two phases of data collection. The first phase was an anonymous online questionnaire consisting of a mix of open-ended free-response questions and closed quantifiable question styles. The second phase of the study consisted of a set of focus groups (Barbour, 2007) designed to validate and enrich the data collected in the questionnaire. As such, discussions were guided by reactions to the results of the first phase of the study. Ethical approval to conduct the case study was obtained from the Faculty Ethics Review Panel for the Institute of Education, Dublin City University.

### Participants

Invitation letters were sent to the principals of the seven identified hospital schools. The letters included plain language statements intended for both the board of management and potential participant teachers that detailed the intended purpose of the study, data collection methods, data retention and storage protocols. Within the plain language statement, participants were informed of their right to not participate in the research and their right to withdraw at any time. Principals were requested to forward the statement together with a link to an anonymous online questionnaire to all the active teachers in their respective hospital schools.

At the time of the research there were 21 active hospital teachers working in the seven hospital schools. Nineteen of these participated in the first phase of the study, an online questionnaire, representing 90% of the hospital teaching population in the Republic of Ireland.

At the end of the questionnaire, participants could volunteer to participate in Phase Two of the study. Fourteen participants volunteered, and 10 of them went on to participate in the second phase of the study, a set of two focus group sessions to delve deeper into the data generated by the questionnaire.

All 19 participants who completed questionnaire had over three years of teaching experience. Eighteen had over five years’ experience, and of these 14 had taught in excess of ten years (see Table 1). Seven of the participants had been teaching less than two years in hospital schools. The remaining 12 had over three years’ hospital school experience, with eight teachers having more than five years and four teachers with in excess of 10 years of hospital teaching experience (see Table 2). This represents a broad spectrum of both teaching in the hospital context and beyond.

**Table 1:** Teaching Experience of Questionnaire Participants.


TEACHING EXPERIENCE	PARTICIPANTS

3–5 Years	1

5–10 Years	4

10+ Years	14


**Table 2:** Hospital Teaching Experience of Questionnaire Participants.


HOSPITAL TEACHING EXPERIENCE	PARTICIPANTS

0–2 years	7

3–5 years	4

5–10 years	4

10+ years	4


Fourteen participants volunteered to also take part in the second phase of the research. Of these, 10 were available to partake in the focus groups. This set of participants were split into two groups, each representing a mix of post-primary and primary trained teachers as well a mix across the individual school sites. All participants had a minimum of one full year of hospital teaching experience. The hospital teaching experience of the focus group participants ranged from one year to more than ten years.

### Procedure

Participants completed an anonymous online questionnaire over a period of two weeks in February 2022. As a part of the questionnaire, they were also asked to complete an informed consent. The questionnaire consisted of seven questions. The first two questions were quantitative and demographic in nature followed by two free-response qualitative questions and a further two quantitative questions. The final question was a reflective qualitative question.

After completing the survey, participants were invited to submit contact information in order to be invited to the second phase of the research. The data generated by the questionnaire were made available to Paul Mahon, the research supervisor, for validation.

Following implementation of the questionnaire, the results were analysed using Braun and Clarke’s six steps (Braun & Clarke, 2013) and compiled into a discussion document, which was used to guide a targeted discussion in the two focus group sessions (see Supplementary File 1). Two focus group sessions lasting one hour each took place in April 2022. Participants were emailed a copy of the discussion document one week prior to the focus group to allow them time to become comfortable and engage with the topics due to be discussed (Barbour, 2007).

The focus groups were conducted over video conferencing software for a number of reasons. First, use of video conferencing software optimised attendance as the seven hospital schools were geographically distant from each other; it would be more respectful of the participants time and less disruptive to their working day. Second, at the time of the survey, healthcare settings were still operating under restrictions due to the COVID-19 pandemic. Since hospital teachers work in close contact with children who are medically vulnerable, it was decided that remote meetings would reduce any potential risk.

Each focus group discussion was audio recorded and then transcribed by the researcher. Both the audio tracks and transcriptions were made available to the research supervisor for validation. Once transcribed, the discussions were thematically analysed using the six steps set out by Braun and Clarke’s (2013) protocol.

- Step One: Initial raw audio and transcript data were engaged with and considered repeatedly to fully familiarise the researcher with the data.
- Step Two: Codes were identified in the transcripts and were reduced and consolidated by identifying patterns.
- Step Three: Initial themes were identified from the compiled codes.
- Step Four: Themes were reviewed to ensure that they reflected the data and made sense to the researcher in the context of the research questions.
- Step Five: The final set of themes were defined and named.
- Step Six: In this paper, the final set of themes are described in the results section and discussed in relation to the research questions in the Discussion section.

## Results

The results of the two phases of the study were synthesised to create a cohesive picture and will be collated under the questions posed by the questionnaire. All data points produced by both the questionnaire and the focus group were included in the analyses; no responses required removal. Data from the questionnaire are labelled Questionnaire Respondent (QR), and data from the focus group are labelled Focus Group Participant (FGP).

### Question 1: What in your opinion are the extra professional development needs of a newly hired hospital teacher?

The following five themes were identified through the qualitative analysis of the answers to this question:

- Working in the Hospital Context
- Educational Needs of Specific Medical Conditions
- Special Educational Needs (SEN) or Additional Educational Needs (AEN)

Training

- Teacher Self-Care and Staff Wellbeing
- Communities of Practice

**Working in the Hospital Context** as opposed to a traditional education setting was raised repeatedly in the responses to Question 1 as being important for the induction of new hospital teachers. It was also a topic of concern in the focus groups, with FGP A4 commenting “*for me coming from mainstream my confidence was knocked because there was so much I didn’t know even after twelve and a half years [teaching]. It was all the medical side of stuff and I felt that just shattered my confidence*.” FGP B2 also pointed to teacher confidence to work in this new environment, *“to just develop the confidence to go in … to the child that looks so unwell.”* FGP A1 went on to point to all of the standard operational procedures in place in the hospital setting that can become a barrier to creating a positive learning experience for new hospital teachers such as personal protective equipment (PPE), isolation protocols, medical terminology, and acronyms.

Another issue that focus group participants felt was important for starting to work within the hospital context was a clear description of the hospital teacher’s role within both the healthcare setting and when working as part of a multidisciplinary team. FGP A1 thought it’s important to set out *“what the role is and … what we are here for.”* FGP A3 agreed, stating *“[we] need to talk about what your role is in general in the hospital not to get too frustrated with yourself when you don’t understand … Loads of days I went home and just went – ‘What did I just do now today?’”* A number of participants felt role ambiguity was an issue that had to be addressed in any form of induction training.

**Educational Needs of Specific Medical Conditions** was another topic frequently mentioned in response to Question 1 of the questionnaire, specifically the *“impact of specific illnesses on educational outcomes”* (QR 14). Further, focus group participants felt that this form of knowledge-based training was vital to hospital teachers. For example, FGP B4 pointed out that newly hired hospital teachers may have no experience *“working alongside children who are undergoing different treatments from medical conditions, chemo-therapies or cystic fibrosis …”* FGP B5 felt that developing this knowledge is “a huge learning curve” for new teachers, but it is important to know “*about the impact of their medical condition on their [ability to learn].”* FGP B4 went on to say it is important to know about the *“different conditions and how to work with these kids … and what the deficits [caused by] their treatment might be.”* FGP A4 agreed that one of the first topics in induction training should be learning about *“all the medical conditions.”*

The theme of **Special Educational Needs or Additional Educational Needs Training** was raised repeatedly by the survey participants. For example, it was stated a number of times that newly appointed hospital teachers should have qualifications or experience teaching children with special educational needs or at least receive training that provides *“an overview of SEN and knowledge of where to access appropriate [support]”* (QR 11). FGP A2 echoed this sentiment, pointing out that *“any child who is in hospital has additional educational needs … they need extra support [both curricular] and in their confidence,”* this is a view also seen in much of the literature (e.g., Closs, 2000). The other participants in the focus group expressed agreement with this stance. This view also ties back to the need for knowledge of how medical conditions and treatments affect the ability to learn, as FGP B5 pointed out their condition *“like children with Sickle Cell disease … it can impair their short term [memory and their] learning capacity.”*

**Teacher Self-Care and Staff Wellbeing** was also a concern raised with regard to hospital teacher induction. Respondents highlighted that training should *“prepare teachers for the emotional challenges of the position”* (QR 13) and allow them to *“develop coping strategies”* (QR 14).

FGP B5 pointed to the *“impact that seeing very sick children can have on somebody when they haven’t been exposed to it before”* and went on to say it is important that hospital teachers are supported in dealing with this. Specifically, FGP A2 commented that we need to be *“conscious of how acute the environment is … families are really, really stressed, children are unwell … it’s a really sensitive environment we are working in and feeling …”* FGP A5 stresses ongoing support and that *“it is really really important for new staff [that they] see what options are available to them in terms of debriefing and supervision.”*

**Communities of Practice** for hospital teachers was the final recurring theme identified in Question 1. New hospital teachers could benefit from attending *“hospital teacher gatherings … developing value connections with other similar professionals to seek and give advice”* (QR 5). New hospital teachers need *“knowledge of the networks that support hospital teachers”* (QR 5) and *“opportunities to explore other hospital school settings and approaches”* (QR 14). A number of participants of the focus group pointed to the importance that CPD *“come from within the community itself”* (FGP B2).

### Question 2: What additional continuous professional development (CPD) should be provided to practising hospital teachers?

The following recurring themes were identified in the questionnaire responses to Question 2:

- Communities of Practice
- Teacher Self-Care and Staff Wellbeing
- Bereavement and Loss
- Additional Needs of Specific Medical Conditions
- Special Educational Needs or Additional Educational Needs Training
- Students With Mental Health Needs
- Post-Primary Curriculum

**Communities of Practice** was one of the most recurring themes for Question 2 of the questionnaire. Respondents cited a number of examples of professional sharing and networking such as *“Teach-Meets”* (QR 12), *“HOPE Congresses”*(HOPE is a European Association of Hospital Teachers) (QR 11 & 14), and *“projects with other hospital schools”* (QR 5, 11, & 14) as important aspects of continuing professional development.

Job shadowing was stressed as one of the most valuable forms of CPD by the focus groups. The idea of peer-to-peer learning was not just for the induction of new teachers. FGP B2 raised the idea that all *“the useful conversations happen when we are finished with the prescribed”* learning. They went on to say that hospital teacher professional development needs to *“come from within the hospital teaching community itself … through shared practice.”* FGP B4 expanded on this later *“we learn so much from attending HOPE conferences … and projects with [European Hospital schools]. You get such fresh ideas when you have those kinds of professional discussions with people in your own [niche] of education.”*

When considering the lack of context-specific CPD, FGP A1 proposed *“the best solution …[could be] coming together ourselves as hospital teachers.”* They went on to mention ways the community might *“share ideas … and ways of doing things … and build stronger connections [and supports] with [other] hospital teachers.”* FGP A5 pointed to when we get together as a community and *“sit and have a chat and share the difficulties and the successes [with hospital teachers],… they can be the most valuable parts of the day”* FGP A4 and A3 agreed that this professional collegial support is important.

**Continuing Teacher Self-Care and Staff Wellbeing** was also a theme that featured strongly in Question 2 of the questionnaire. Questionnaire respondents called for *“training on self-care for the teacher”* (QR 3) and *“CPD concerning teacher wellbeing”* (QR 4) together with *“Group counselling/discussion sessions”* (QR 7). The focus group participants also focused on this particular concern for this issue. For example, FGP A3 raised awareness *“of the emotion that is all around us in the [work environment]. It can be very much charged emotion.”* FGP A4 brought up an example of how hospital teachers *“constantly walk down corridors, you see parents embrace, you don’t even know them, but you know something tragic has happened …”* FGP B5 stated that schools *“need to make a conscious effort to support everybody in taking care of themselves.”*

One of the potential CPD strategies identified by the focus group participants to address this theme was emotional intelligence training and interpersonal communication skills. FGP A5 stated, *“I think communication-interpersonal skills are really important and they should be worked on … constantly;”* they went on to say *“hospital teaching … possibly attracts people who have … good interpersonal skills … and good emotional intelligence but [you need] to maintain that and build on that.”* FGP A4 and A5 both agreed that *“debriefing and supervision, it’s so important.”* They stressed that it is important that this is an ongoing support *“because it is when you’re in the middle of something … that you need it most.”*

Dealing with **Bereavement and Loss** was mentioned as a distinct category within the theme of teacher self-care and staff wellbeing theme. Questionnaire respondents mentioned *“supported skills in coping with bereavement”* (QR 2). They looked for *“CPD on dealing with bereavement and loss”* (QR 5) and *“CPD in dealing with grief”* (QR 19). In the focus groups, FGP B5 raised the topic of dealing with *“bereavement … once you are in this setting [you need to be mindful] of the impact of seeing very sick children … or if you do lose a pupil … a lot of us are good at talking about how we are feeling … but … not everybody is.”*

**Additional Educational Needs of Specific Medical Conditions** was another recurring theme raised in the questionnaire responses. Respondents called for training in specific medical conditions *“in the form of medical education sessions from professionals in the various disciplines such as psychology, oncology, cardiac, etc.”* (QR 8). The focus group participants focused on this topic mostly in relation to newly hired hospital teachers but recognised, as FGP A1 pointed out, that there is a need for all hospital teachers to keep *“updating and updating.”* FGP B5 remarked that while this is an important topic for new hospital teachers, they are *“still blown away when they [continue to] learn [how medical conditions] affect a child’s education … [or what can] impair their … capacity to [learn].”* FGP B4 pointed out that *“hospital treatments can cause additional educational needs, I have come across children who’ve had deficits caused by their treatments … it’s important [for hospital teachers] to have … a vast [knowledge base]… to pull from to cater for the kid that’s in front of you.”*

Within this theme respondents highlighted **Students With Mental Health Needs** specifically. They looked for training on *“how to support students [admitted] under mental health”* (QR 3) and *“CPD on mental health issues such as eating disorders, overdose and deliberate self-harm”* (QR 5). Participants of the focus group also highlighted this as an area that required development. For example, FGP B1 pointed to a “*surge in children presenting with anxiety disorders and mental health issues … need to ensure we are well equipped to [deal with them]…, that we have gotten the right CPD training.”* This sentiment was echoed by FGP B3, who was looking for advice on *“how to approach mental health issues raised by some curricula with these students.”* FGP A3 commented that for working with *“students with psychiatric needs, I feel I really need to improve or hone my communication skills and interpersonal skills …”*

Respondents to the questionnaire suggested **Special Educational Needs or Additional Educational Needs Training** as important for practising hospital teachers. Specifically, they suggested *“CPD in different categories of special ed”* (QR 18), *“AEN teaching and learning supports”* (QR 9), and training in *“Level 1 and Level 2 at post-primary level”* (QR 2). Both FGP B2 and FGP A1, in separate focus groups, pointed out that hospital schools represent a “*cross-section of the country and … the educational field*,” so we see the full range of special needs from students with *“dyslexia or dyscalculia … to students with sensory issues [to students who are] nonverbal.”* Similarly FGP B5 referred to the *“vast number of children”* who present to hospital schools, many of whom have special educational needs *“even those with profound needs.”* They felt that despite their qualifications, they “*need to up-skill again*.” FGP B4 went on to say there is *“such a broad spectrum”* of students that hospital teachers work with and that even if you have training and experience in SEN, it is usually in one specific area *“and not all of them*. As an example, they cited teacher who *“may feel a bit of an expert in autism”* but is presented with a *“child who has a visual or hearing impairment”* and may now *“feel a shortfall in their training”* (FGP B4).

**The Post-Primary Curriculum** was raised as a CPD concern by the respondents of the questionnaire. “*CPD on Junior Cycle [Irish post primary curriculum for students aged 12 to 15] subjects beyond your own subject*” (QR 3) and “*CPD in RACE and DARE and other access programs*” (QR 2) were both cited as necessary. Focus group participant FGP B3 explained that post-primary teachers have one or two *“base subjects; it’s good in one way to do [other] subjects, but at the same time it’s challenging… so I professional development in terms of that.”* FGP B5 stated that *“it takes a lot of confidence to try and plan and prepare for [subjects beyond your base one or two subjects].”* FGP A1 pointed to the fact that post-primary *“students [might be] doing eleven subjects and I see them for half an hour. How am I going to replicate their school experience?”* FGP B1 talked about smaller hospital schools where CPD in post-primary subjects is needed for primary trained staff. *“I’ve just felt an obligation to make sure I am on the top of my game with regards to the core [post-primary] subjects as [both teachers] are primary trained.”*

### Question 3: Please rate the following proposed continuous professional development topics on a scale of 1 to 7, where 1 is least relevant and 7 is most relevant to the context of the hospital teacher

The quantitative results of this question are listed in Table 3.

**Table 3:** Questionnaire Respondents’ Perceptions of the Relevancy of proposed CPD topics.


CPD TOPIC	RELEVANCE (SCALE 1–7)

Teaching Additional Educational Needs	6.7

Communication & Interpersonal Skills	6.7

Resilience Skills	6.5

Emotional Intelligence	6.4

Knowledge of Specific Medical Conditions	6.4

Bereavement & Loss	6.3

IT Skills & Digital Literacy	5.7

Mindfulness	5.7

Curricular Topics	5.7

Behaviour Management Skills	5.5

Leadership Skills	5.3

Admin & Record Keeping	4.8


*Note*. Questionnaire respondents were asked to score the relevancy of proposed CPD topics on a Likert scale from 1 to 7 (1 = the least relevant, and 7 = the most relevant).

### Question 4: Drag the following possible CPD topics into the order of priority to be included in professional development for hospital teachers

The quantitative results of this question are listed in Table 4 below.

**Table 4:** Questionnaire Respondents’ Perceptions of the Priority of CPD Topics for Hospital Teachers.


PRIORITY	CPD TOPIC	SCORE (SCALE 1–12)

1	Communication & Interpersonal Skills	3.1

2	Teaching Additional Educational Needs	3.6

3	Emotional Intelligence	4.3

4	Knowledge of Specific Medical Conditions	4.8

5	Bereavement and Loss	4.9

6	Resilience Skills	6.0

7	Curricular Topics	6.5

8	IT Skills & Digital Literacy	7.7

9	Mindfulness	8.2

10	Leadership skills	9.2

11	Behaviour Management Skills	9.2

12	Admin & Record Keeping	10.4


*Note*. Questionnaire respondents were asked to score the priority level of CPD topics for hospital teachers (where 1 = highest priority and 12 = least priority).

The qualitative responses to Questions 3 and 3 follow.

**Communication & Interpersonal Skills** was one of two topics deemed both to have the most relevancy by respondents and to be of highest priority (see Tables 3 and 4). FGP B2 felt that communication & interpersonal skills scored so highly as “*[Multi Disciplinary Team] (MDT) teams can be very intimidating for teachers … [teacher] observations are … very valuable … so communicating is really, really important.”* FGP B5 agreed and added, *“communication and interpersonal skills … with the students … we’re nearly salespeople … trying to make them more comfortable … make parents more comfortable and find ways to get them to attend.”* FGP A5 agreed that working with children on the wards *“can be very daunting … to go in and introduce yourself to a parent and student … So I think communication skills, we should be working on them constantly.”* FGP B1 echoed both sentiments, adding *“there’s a lot of reservations, hesitancy about … attending … we need to create the right atmosphere … to engage with them.”* FGP A4 said, *“we’re … such a part of a team … sharing students … sharing what’s being taught … we’re so close knit together that interpersonal skills are invaluable.”* They continued, *“There’s so many interactions with secretaries, nurses, cleaners … constantly talking to people … completely different to mainstream, in my opinion.”*

**Teaching Additional Educational Needs** was the second CPD topic deemed most relevant and of highest priority by respondents. FGP A5 stated they *“have seen the need … to upskill in [the SEN] area … pupils coming from [special] schools … with very positive educational experiences … it’s important to try to replicate that if we can.”* FGP A2 pointed out *“any child who is in hospital has additional educational needs … even a child who is going to mainstream … they need extra support.”* FGP A4 felt the topics scored high because *“[students from special schools] pose the most challenge for me … it’s an area I want to do better at.”* FGP B5 suggested *“it is rated highly [as there is] still not a big enough focus in teacher’s college on SEN.”* FGP B4 stated *“the hospital teaching community … have come from different experiences and … there’s such a broad spectrum of [special educational needs].”*

Professional Development in **Emotional Intelligence** featured strongly in the list of priorities and relevance. FGP A3 explained *“that [we are] aware of the emotion that is all around us here and it is very much charged emotion.”* FGP A4 cited an example of *“seeing two parents embrace, you don’t even know them, but you know something tragic has happened.”* FGP A2 *“would love to learn more about … dealing with someone who is in a very stressed environment … The main thing is I don’t want to do any harm.”* FGP A5 agreed, citing their mantra of *“go gently … because you don’t know what you are meeting at a bedside day to day. It extends to families … staff … even our own teacher”* They continued that hospital teachers need *“good emotional intelligence … to maintain that and build on tha t… and equip our new teachers [with] training in that to prepare them.”*

FGP B5 was surprised that **Leadership Skills** did not score higher in terms of relevancy and priority (see Tables 3 and 4). *“I think as a classroom teacher, you’re the leader, it’s your realm … maybe it’s because we work as such a collaborative group in the hospital school setting … that’s one that surprised me, the others not so much.”* Participants displayed a lack of surprise at **Admin & Record Keeping** being the least relevant and lowest priority. FGP B2 proclaimed it *“a principal’s problem.”*

## Discussion

A previous scoping review of the international literature pointed to the need for context-specific training for hospital teachers and a number of possible content areas that might prove valuable for hospital teachers (McNamara, 2022). The goal of the present study was to identify and explore the continuing professional development needs of hospital teachers in Ireland. To that end, the study investigated the perceptions of teachers working within the context in Ireland between February and May 2022.

The findings of this qualitative study provide clear evidence that Irish hospital teachers share a consensus of perceptions on the professional development requirements for their unique educational context. Many of the recurring themes raised by the teachers in the questionnaire phase of the research mirror those in the literature (McNamara, 2022).

### Hospital Teacher Induction

The participants of the study perceived the following professional development topics to be necessary to put in place for the induction phase of newly hired hospital teachers:

- Working in the Hospital Context
- Educational Needs of Specific Medical Conditions
- Special Educational Needs (SEN) or Additional Educational Needs (AEN)

Training

- Teacher Self-Care and Staff Wellbeing
- Communities of Practice

Three of these topics (hospital context, specific medical conditions, and teacher self-care) also feature strongly in the international evidence (McNamara, 2022). SEN or AEN training and Communities of Practice were two additional topics identified by the present study.

Special educational needs (SEN) or additional educational needs (AEN) training was identified by this study as being important for teachers entering the hospital context. Although many of the children admitted as inpatients to hospital come from the world of mainstream education, it is recognised that while hospitalised due to their illness or their treatment, they have additional educational needs (Closs, 2000). Participants of this study acknowledged that finding and also pointed to the fact that hospital schools represent a cross-section of society, so hospital teachers are called upon to work with the entire range of SEN regularly during their careers.

With regard to working in hospital schools, in addition to the practical considerations of working in this context, participants raised concerns around role ambiguity given the vast differences between the hospital teaching environment and traditional classroom settings. It was perceived that induction must provide for a clear role description and realistic expectations of the daily routines for the new hospital teacher.

The educational needs of specific medical conditions is a knowledge-base requirement that is specific to hospital teaching. This information is currently acquired via informal and ad-hoc communication with medical teams in the various hospital sites. Participants of this study hoped to see it become more formal and systematic. It is important that newly hired hospital teachers begin to accumulate this knowledge as soon as possible after entering service.

Teacher self-care and staff wellbeing is recognised in the literature as an important aspect of professional development for hospital teachers (Benigno et al., 2018; Hen, 2020; Keehan, 2021; Shore, 2019). The participants of this study also highlighted this as a concern. They called for this to be implemented early during the induction phase and suggested that newly hired staff be educated about all of the support options that are available to them.

Participating in communities of practice was highlighted by this study as a professional development support that should start during the induction phase. Opportunities for networking and job shadowing with professionals in the same educational niche would help new entrants begin to understand and define their role as hospital teachers.

### Continuing Professional Development for Hospital Teachers

This study identified seven themes or topics that are important for the practising hospital teacher to engage with as part of their ongoing professional development:

- Communities of Practice
- Teacher Self-care and Staff Wellbeing
- Bereavement and Loss
- Additional Needs of Specific Medical Conditions
- Special Educational Needs or Additional Educational Needs Training
- Students With Mental Health Needs
- Post-Primary Curriculum

Developing and working with communities of practice featured strongly in the questionnaire responses and focus discussions in this study. The participants perceived this to be the most valuable source of context-specific professional development, at a local, national, and even international level – from ongoing job shadowing and team teaching in house to networking opportunities and projects within the Irish hospital teaching community and beyond to our European partners in organisations such as the Hospital Organisation of Pedagogues in Europe (HOPE) and through EU funded projects such as the Erasmus Plus programme.

Ongoing teacher self-care and staff wellbeing was also repeatedly mentioned by participants. The literature is also strong on this (Benigno et al., 2018; Harris & Farrell, 2004; Hen, 2020; Keehan, 2021; Shore, 2019). It is important that these professional development supports are in place throughout the teaching calendar as the support is needed while on the job and should not be relegated to summer courses or provided sporadically in isolation. It was suggested it should be provided through both a debriefing/supervision counselling format and as skills-based training. Training that specifically deals with bereavement and loss was also raised by participants as being an important component of this area.

While important for newly trained hospital teachers, training on students’ additional educational needs due to specific medical conditions is also of concern for all practising hospital teachers. This study highlighted that hospital teachers need to stay up-to-date on the effects of certain illnesses and treatments on children’s propensity to learn. This helps them to differentiate and adapt lessons for specific cohorts of children.

This study also pointed to the need for practising hospital teachers to continue to up-skill on special educational needs or additional educational needs. As hospital teachers must be able to work with students across the full spectrum of needs, it is important for them to have a broad education in SEN provision. The study also highlighted hospital teachers’ need to engage with training for working with students with mental health needs, as this particular cohort of children was mentioned as providing a challenge to the participants of the study.

The study highlighted a division between the professional development needs of primary and post-primary trained teachers. Unlike primary teachers, traditionally post-primary teachers specialise in one or two curricular subjects. As Irish hospital schools are small multi-level schools, they cannot employ the full spectrum of post-primary trained teachers. Post-primary hospital teachers must, therefore, provide education in subjects that are often beyond their training and qualifications. On the other hand, some smaller hospital schools only have primary trained teachers. In these contexts, the primary teachers must teach the post-primary students and therefore beyond their own qualifications. There is an expressed need here for professional development in the post-primary curriculum, particularly in the core subjects.

### Relevancy and Priorities

The study also asked the participants to score a set of proposed professional development topics according to their contextual relevancy and to ascribe a priority level.

Both Communication & Interpersonal Skills and Teaching Additional Educational Needs were perceived as the most relevant and of the highest priority for hospital teachers. This is understandable as unlike teachers working in a classroom setting, hospital teachers work in a highly complex web of professional relationships. A large community of medical professionals, parents, students and support staff must be engaged with on a daily basis. This social complexity is also recognised in the literature (Avalos & Fernandez, 2021; McNamara, 2022). Similarly, as previously mentioned, hospital teachers in the current study perceived all hospitalised students as having some degree of additional educational need almost by definition. This perception is also acknowledged in the literature (Closs, 2000).

### Practical Implications of the Research

There is an identified need for quality induction courses for newly hired hospital teachers. It is clear that hospital teachers working in Ireland in 2022 perceive a need for targeted contextualised professional development. These forms of learning opportunities do not currently exist for this population of teachers.

The hospital teachers who participated in this case study identified and prioritised a set of potential topics. Thus, the results of this study can inform the creation of a curriculum for future hospital teaching professional development in Ireland. It is also hoped that while the findings of this case study are limited to the Irish context, they may provide useful information that is applicable in similar hospital school communities further afield.

## Conclusion

This qualitative case study explored possible topics for the provision of targeted contextualised professional development opportunities for Irish hospital teachers. The study aimed to identify and document the perceptions of currently active hospital teachers on what their professional development needs were. The international evidence showed that there is a clear need to provide context-specific training and learning opportunities to hospital teachers (McNamara, 2022). Nevertheless, no formal induction courses or continuing professional development courses that target hospital teachers are available in Ireland at the time of writing. The findings of this study reinforce this consensus view of the literature. Participants of the study clearly vocalise a need for context specific training in the Irish hospital school setting.

The findings identify a number of context-specific needs that it is important to meet to ensure Irish hospital teachers’ professional development and wellbeing. Respondents in this study set out their priorities with regard to addressing these needs starting with developing interpersonal and communication skills and establishing a broader knowledge and skill base for working with children with special educational needs. Further, participants proposed communities of practice, peer-to-peer learning and job shadowing as the most effective methodology for providing professional learning opportunities in the hospital school context.

### Limits of the Study

As with any research using case study methodology, the findings of this study are limited to the temporal and geographical space in which it was conducted. The findings represent the perceptions of hospital teachers working in the seven Irish hospital schools between January and May 2022.

While the sample size of the questionnaire was small, with 19 hospital teachers responding, this nevertheless represents 90% of the population. Similarly, while only 10 hospital teachers participated in the focus group phase, this represents 48% of the entire population of Irish hospital teachers.

### Implications for Further Research

The study has identified a number of professional learning topics perceived as important for supporting the work of Irish hospital teachers between January and May 2022. It would be a valuable to repeat the study periodically within the Irish context to provide longitudinal data. Such longitudinal research could explore how the provision of identified training impacts the Irish hospital teachers and measure its effectiveness in meeting the needs of this cohort of teachers. In addition, conducting similar studies across populations of hospital teachers outside of Ireland would allow for comparative analysis of a number of international contexts. This would help to inform best practice, globally, in the professional development of hospital teachers.

## Additional File

The additional file for this article can be found as follows:

10.5334/cie.123.s1 Supplementary File 1. Focus Group Discussion Document.pdf.
