# Supplementary material for: The Professional Development Needs of Hospital Teachers in Ireland: An Exploratory Case Study
Source: Contin Educ. 2024 Apr 15;5(1):50–65. doi: 10.5334/cie.123 (PMC11104299; doi:10.5334/cie.123)
Supplement: Supplementary File 1. — Focus Group Discussion Document.pdf. [file cie-5-1-123-s1.pdf]

An exploratory case study of the perceptions of practicing Irish hospital teachers regarding the professional development needs required for working in the Irish hospital school context.

SURVEY RESULTS

The following booklet presents the results of the recently completed survey.

Open ended answers are presented with the identified emerging themes with some quoted examples from the data.

Numerical questions have been presented in the form of a table

What, in your opinion, are the extra professional development needs of a newly hired hospital teacher?

SEN or AEN Training

"An overview of SEN and where to access appropriate support/ training"

"Post-grad, CPD or experience teaching SEN"

"Training on teaching children with additional needs"

"Training in SEN - Lámh, basic sign language etc"

Teacher Self-care and Staff wellbeing

"emotional challenges of the position

"Employee assistance, wellness and opportunities for developing coping strategies"

"Self care for the new teacher"

Communities of Practice

"Annual hospital teacher gathering would help develop professional value connections to seek help and give advice"

"Knowledge of support networks for hospital teachers. eg HOPE and current Irish hospital teachers group"

"Opportunities to work with other Irish hospital schools, explore settings and approaches. Attend HOPE conferences"

Needs of Specific Medical Conditions

"What works well with children with different medical needs"

"medical condition specific conferences"

"Knowledge of educational needs within particular areas"

"impact of specific illnesses on educational outcomes"

"Training on different medical conditions and how it affects children and teens in the classroom"

The Hospital Context

"Working as part of a multi-disciplinary team"

"Child protection specific to bedside teaching"

"HSE training - Hand Hygiene, Fire Safety, First aid etc"

"What resources can be used in different hospital settings? eg. isolation etc"

"Orientation - Hospital maps, roles of HSE staff in the hospital, colour coded uniforms etc"

What additional CPD should be provided to practicing hospital teachers?

SEN or AEN Training

"CPD in different categories of special ed especially working with non-verbal children"

"AEN teaching and learning support"

"Training in Spec. Ed. "

"Level 1 & Level 2 at post-primary level."

Teacher Self-care and Staff wellbeing

"Training on self care for the teacher"

"CPD concerning teacher wellbeing"

"Staff wellbeing activities…Group counselling/discussion settings."

"setting clear boundaries… tied to this is maintenance of our own self care"

Bereavement and Loss

"support and skills in coping with bereavement"

"CPD on dealing with bereavement and loss"

"CPD in dealing with grief"

Communities of Practice

"Teach-Meet opportunities to contact fellow hospital teachers and share ideas"

"Opportunities to work with other hospital schools… attending HOPE conferences"

"inter-hospital school project opportunities… developing a research culture"

"training through sharing and collaboration with other hospital teachers"

Needs of Specific Medical Conditions

"CPD for specific medical conditions. Cardiac, sickle cell burns etc"

"tailored CPD in the form of medical education sessions from professionals in the various disciplines eg. psychology, oncology, cardiac, burns etc"

"training in particular areas such as neurosurgery, renal, etc"

Students Mental Health

"How to support students under Mental Health"

"CPD on mental health issues such as eating disorders, overdose, and deliberate self harm"

"Training in the area of mental health"

Post-Primary Curriculum

"Junior Cycle subject CPD workshops for hospital schools"

"CPD on JCT subjects beyond your own subject"

"CPD in RACE & DARE and other access programs"

Please rate the following proposed CPD topics on a scale of 1 to 7, where 1 is least relevant and 7 is most relevant to the context of the hospital teacher.

	CPD Topic	Relevance (Scale 1-7)
1	Teaching Additional Educational Needs	6.7
2	Communication & Interpersonal Skills	6.7
3	Resilience Skills	6.5
4	Emotional Intelligence	6.4
5	Knowledge of Specific Medical Conditions	6.4
6	Bereavement & Loss	6.3
7	I.T. Skills & Digital Literacy	5.7
8	Mindfulness	5.7
9	Curricular Topics	5.7
10	Behaviour Management Skills	5.5
11	Leadership Skills	5.3
12	Admin & Record Keeping	4.8

Drag the following possible CPD topics into the order of priority to be included in professional development for hospital teachers

	CPD TOPIC	Priority (Scale 1-12)
1	Communication & Interpersonal skills	3.1
2	Teaching Additional educational needs	3.6
3	Emotional Intelligence	4.3
4	Knowledge of Specific Medical Conditions	4.8
5	Bereavement and loss	4.9
6	Resilience skills	6.0
7	Curricular topics	6.5
8	I.T. Skills & Digital Literacy	7.7
9	Mindfulness	8.2
10	Leadership skills	9.2
11	Behaviour Management Skills	9.2
12	Admin & Record Keeping	10.4

On reflection, are there any additional areas of CPD that you would like to suggest as important to the role of hospital teacher?

Teacher Self-care and Staff wellbeing Emotional Intelligence, resilience and bereavement training

Digital Literacy, Digital tools and Assistive technology

Collaboration & Team Building Skills

Students Mental Health

Special Consideration of Curricular CPD for Post-Primary Teachers
